# Case report of a mature cystic teratoma in the tail of the pancreas in a 54-year-old male

**DOI:** 10.3389/fmed.2026.1780193

**Published:** 2026-03-30

**Authors:** Zheng-min Mo, Jing He, Gang Ji, Yi-hui Wang, Bin Zhou, Lei Wang

**Affiliations:** 1 Department of Radiology, Mianyang Wanjiang Eye Hospital, Mianyang, China; 2 Department of Pathology, The Third Hospital of Mianyang, Sichuan Mental Health Center, Mianyang, China; 3 Department of Radiology, Mianyang Central Hospital, Mianyang, China

**Keywords:** case report, computed tomography, dermoid cyst, mature cystic teratoma, pancreas

## Abstract

**Background:**

Pancreatic dermoid cyst (also referred to as mature cystic teratoma) is a rare, histologically well-differentiated benign germ cell tumor. Preoperative definitive diagnosis remains challenging due to the absence of specific imaging features and reliable serum tumor markers.

**Case description:**

We present a case of a 54-year-old male patient in whom a cystic low-density lesion, measuring 3.08 cm × 2.82 cm × 2.82 cm with predominantly well-defined margins and relatively homogeneous density, was incidentally detected in the pancreatic tail during a routine physical examination. Preoperative imaging assessment indicated a cystic neoplasm; however, its benign or malignant nature could not be definitively ascertained. The initial preoperative plan under general anesthesia included laparoscopic exploration and abdominal mass resection, with a contingency plan for combined distal pancreatectomy and splenectomy. During the operation, the chief surgeon observed with the naked eye that the mass had a complete capsule and was only closely adhered to the tail of the pancreas, making it easy to resection. Therefore, the surgery was adjusted to laparoscopic pancreatic lesion resection combined with percutaneous abdominal drainage. Postoperative pathological examination confirmed it to be a cystic mature teratoma. Given the potential malignant risk associated with such lesions, regular postoperative imaging follow-up was scheduled to evaluate treatment outcomes. To date, the patient has completed over 10 months of postoperative follow-up with no evidence of recurrence.

**Conclusion:**

Pancreatic dermoid cysts are extremely rare, and their diagnosis depends on postoperative pathological examination. Although preoperative confirmation remains challenging, this retrospective study underscores the diagnostic difficulties in imaging-based evaluation of pancreatic cystic lesions and systematically summarizes the current research advances and conceptual framework regarding these uncommon tumors. The findings may help prevent unnecessary extensive surgical resections resulting from misdiagnosis of pancreatic primary cystic neoplasms.

## Introduction

Mature cystic teratoma (MCT) is a rather common benign germ cell tumor, which is often diagnosed incidentally (1). MCT is composed of well-differentiated structures from the ectoderm, mesoderm and endoderm (2). Ectodermal tissue and sebaceous material are common and ubiquitous in almost all cases. The most common ultrasonic manifestation is a cystic echo with a hyperechoic nodule, which is composed of hyperechoic sebaceous material and calcification (3). It can occur in women of different age groups, but is more common in women of childbearing age (4). It is frequently found in the gonads and sacrococcygeal region (5), especially in the pediatric population, but its occurrence in the pancreas is extremely rare. The possible causes of this type of tumor remain unclear. Risk factors include late menarche, long-term irregular menstruation, a history of cystic teratoma, few pregnancies, infertility, excessive alcohol consumption and lack of exercise (6).

Pancreatic teratoma is an extremely rare primary tumor. Because of its well-differentiated nature and the predominance of ectodermal components, it is often referred to as a dermoid cyst. It was first described by Kerr (7), Jacobs and Dinsmore (8). The reported cases in the existing literature are relatively rare, and clinical experience is still insufficient. It mainly occurs in young patients, with an average age of 36.9 years, and woman have a slight advantage (Table 2). The most common location of the tumor is the head of the pancreas (31%), followed by the body (29%), and 24% of the cases involve the tail (Table 2). The average size of the tumor is approximately 8 cm (ranging from 2.2 to 22 cm) (Table 2). Clinical manifestations and physical examinations are usually non-specific (9). Previous literature reports indicate that abdominal pain is the most common clinical manifestation (44.0%), followed by asymptomatic incidental findings (25.9%), with abdominal masses ranking third (19.0%) (Table 2). Surgical resection is the preferred diagnostic method and the main treatment approach (10). When surgery is contraindicated or the patient refuses it, non-steroidal anti-inflammatory drugs (NSAIDs) can be used to reduce the size of the DC or slow its growth (11). The prognosis is good after complete surgical resection, with an overall 5-year survival rate approaching 100% (12). However, in some rare cases, they can progress to a malignant phenotype (13).

In this report, we present a rare case of a mature cystic teratoma in the tail of the pancreas. To the best of our knowledge, this is the 59th case reported in the literature (Table 1), highlighting its rarity. This case underscores the challenges in preoperative imaging diagnosis of pancreatic dermoid cysts, thereby aiding in the avoidance of unnecessary surgical resection resulting from misdiagnosis as primary cystic neoplasms of the pancreas.

**TABLE 1:** Reported cases of dermoid cyst of the pancreas.

Number	First author, year	Age (years)/gender	Maximum diameter (cm)	Location	Symptoms	Surgery	Recurrence or metastasis or malignant transformation
1	Kerr (7)	55/F	/	Head	Abdominal mass	Pancreaticcystectomy	/
2	Dennins (22)	33/F	/	Head	Back pain and abdominal mass	Marsupialization	No
3	Decourcy (23)	2/F	/	Body	Emesis, vomiting	Pancreatic cystectomy	/
4	Hoang-Su (24)	/	/	/	Back pain	Pancreatic cystectomy	/
5	Bittner and Sarrazin (25)	2/F	/	Head	Liver failure	Pancreatic cystectomy	/
6	Iovchev (26)	8/M	/	Body	Emesis and abdominal pain	Drainage	/
7	Pomosov et al. (27)	6/M	/	Tail	Abdominal pain	SPG	/
8	Tobik et al. (28)	34/F	/	/	Abdominal pain	Internal drainage	/
9	Assawamatiyanont and King (29)	11/F	9	Body	Abdominal mass	Pancreatic cystectomy	/
10	Lázaro da Silva and Moreno Júnior (30)	21/M	>3	/	Abdominal mass and emesis, constipation	Pancreatic cystectomy	No
11	Vermeulen et al. (31)	46/M	–	Body	Asymptomatic	SPG	/
12	Mester et al. (32)	25/F	8	Head	Abdominal pain	Pancreatic cystectomy	No
13	Jacobs and Dinsmore (8)	57/F	6.5	Body	Abdominal pain and weight losing	Pancreatic cystectomy	/
14	Markovsky and Russin (33)	53/F	20	Body	Abdominal pain	Pancreatic cystectomy	/
15	Iacono et al. (34)	26/F	12	Head	Abdominal pain and fever	DPC	No
16	Kraimps et al. (35)	/M	/	/	/	Pancreatic cystectomy	/
17	Das et al. (36)	4 months/F	9.5	Body-tail	Abdominal mass	Pancreatic cystectomy	/
18	Lushpai (37)	/	/	/	/	/	/
19	Fernandez-Cebrian et al. (38)	74/M	10	Body	Backache pain	SPG	No
20	Strasser et al. (39)	44/M	7	Head	Abdominal pain	/	Recurrence
21	Yu et al. (40)	2/M	12	Head-body	Abdominal mass	Pancreatic cystectomy	/
22	Salimi et al. (41)	16/M	/	Head	Jaundice and weight loss	Choledochoduodenostomy	/
23	Seki et al. (42)	60/F	22	Body	Asymptomatic	Middle pancreatectomy	/
24	Seki et al. (42)	57/M	5.5	Body	Asymptomatic	Pancreatic cystectomy	/
25	Koomalsingh et al. (43)	52/M	3.2	Tail	Abdominal pain	Pancreatic cystectomy	No
26	Rivkine et al. (44)	45/F	5.5	Head	Abdominal pain	Pancreatic cystectomy	/
27	Tucci et al. (45)	64/M	8.5	Tail	Asymptomatic	Distal pancreatectomy	/
28	Yoon et al. (46)	57/M	/	Head	Abdominal pain	/	/
29	Zhang et al. (47)	67/M	4.6	Body	Asymptomatic	SPG	/
30	Mateos et al. (48)	39/F	8.5	Body	Abdominal pain	SPG	No
31	Scheele et al. (49)	40/M	6.4	Head-body	Abdominal pain	DPC	/
32	Badia et al. (50)	43/F	15	Body	Abdominal pain and emesis	Distal pancreatectomy	/
33	Urata et al. (51)	58/M	2.8	Tail	Back pain	Distal pancreatectomy	/
34	Degrate et al. (1)	61/M	3.5	Head	Asymptomatic	DPC	No
35	Lane et al. (52)	63/M	5.7	Body	Abdominal pain	Pancreatic cystectomy	No
36	Albayrak et al. (53)	20/F	10	Head-body	Abdominal pain	Pancreatic cystectomy	Died from postoperative complication (massive intra-abdominal hemorrhage and hypovolemic)
37	Campani et al. (54)	59/M	8	Tail	Asymptomatic	Distal pancreatectomy	/
38	Lyons et al. (55)	35/M	2.5	Tail	Asymptomatic	SPG	/
39	Wang et al. (56)	11 months/F	15	Body	Abdominal mass and anorexia	Pancreatic cystectomy	14 months after the second surgery, tumor recurrence
40	Lee et al. (57)	54/M	4.5	Tail	Asymptomatic	Distal pancreatectomy	No
41	Ahmed et al. (17)	65/M	7	Body-tail	Abdominal pain and early satiety	Distal pancreatectomy	/
42	Chakaravarty et al. (58)	41/M	4.8	Body	Abdominal pain and abdominal mass	Pancreaticcystectomy	/
43	Ofori et al. (59)	49/M	5.7	Tail	Abdominal pain	FNA	/
44	Sasaki et al. (60)	12/F	7	Head	Abdominal mass	Pancreas head was made, and Pancreatic cystectomy	No
45	Saikaly et al. (61)	29/M	/	Head	Abdominal pain	Whipple and total pancreatectomy	/
46	Li et al. (62)	36/F	20	Tail	Abdominal pain	Partial pancreatectomy, left colon resection, partial gastrectomy, duodenectomy, as well as inferior vena cava and renal vascular repair	No
47	Kim and Koh (63)	53/M	4.4	Tail	Asymptomatic	Distal pancreatectomy	/
48	Wang et al. (64)	13 months/M	12	Head	Abdominal mass	PPPD	No
49	Cemeroglu et al. (65)	6 month/F	16.5	No connection to the pancreas	Hyperinsulinemic hypoglycemia	Pancreatic cystectomy	No
50	Zhou et al. (66)	23/F	8.3	Head	Abdominal pain	Roux-en-Y choledochojejunostomy with gastrojejunostomy was performed, and pancreatic cystectomy	No
51	Munoz et al. (67)	72/F	7.5	Body-tail	Asymptomatic	SPG	No
52	Djokic et al. (68)	33/F	8.5	Head	Abdominal pain	PPPD	Neuroendocrine tumor occurred.
53	Tasis et al. (9)	29/F	17	Body-tail	Difficulty in swallowing, nausea, abdominal pain, shortness of breath and vomiting.	SPG	No
54	Kori et al. (69)	39/F	18	Tail	Asymptomatic	Distal pancreatectomy	No
55	Al Jada et al. (70)	30/M	8.8	Head	Asymptomatic	Whipple	No
56	Tee et al. (71)	55/M	5.0	Tail	Asymptomatic	SPG	/
57	Liu et al. (72)	/	/	/	/	/	No
58	Liu et al. (72)	/	/	/	/	/	No
59	Our case 2026	54/M	3.5	Tail	Asymptomatic	Pancreatic cystectomy	No

SPG, splenopancreatectomic gauche; DPC, duedunopancreatectomy cephalic; PPPD, pylorus preserving pancreaticoduodenectomy.

## Case presentation

**FIGURE 1:**
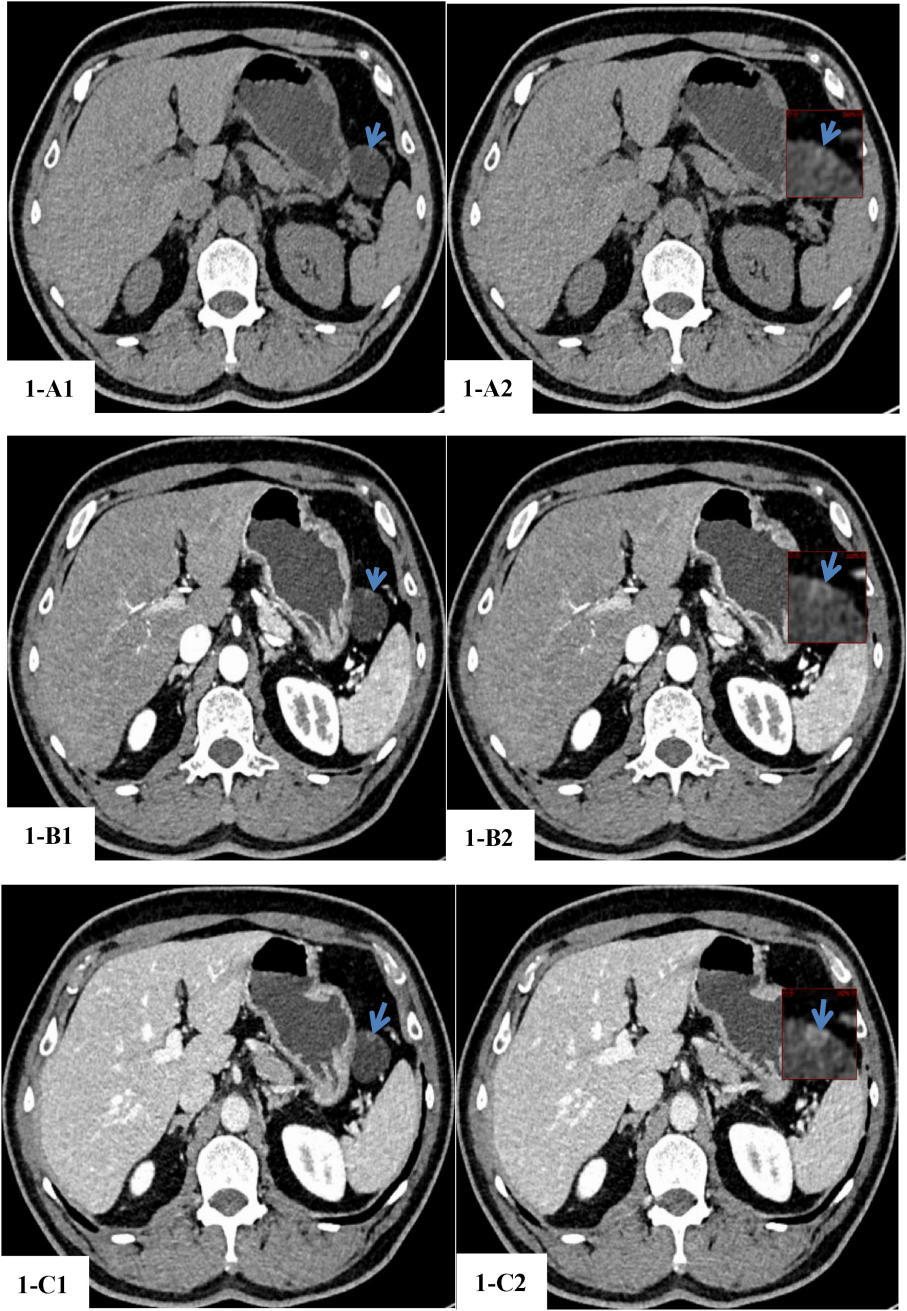
CT images of a space-occupying lesion in the tail of the pancreas. **(1-A1)** High-resolution non-contrast CT scan with thin transverse sections: the lesion is located in the pancreatic tail, appears ovoid, demonstrates hypodensity (CT value: −12 HU), has maximum dimensions of approximately 3.08 cm × 2.82 cm × 2.82 cm, and corresponds to the region indicated by the blue arrow. **(1-A2)** High-resolution non-contrast CT scan with thin transverse sections: upon magnification, a high-density nodular opacity with a CT value of 17 HU is visualized within the cyst wall, and corresponds to the region indicated by the blue arrow. **(1-B1**) Thin-section transverse CT scan of the arterial phase following contrast enhancement: the CT value of the hypodense region within the tumor lesion is approximately −18 HU, and corresponds to the region indicated by the blue arrow. **(1-B2)** Thin-section transverse CT scan of the arterial phase following contrast enhancement: upon magnification, a mural nodule with a diameter of about 0.6 cm was visible at the anterior margin of the lesion, which showed significant enhancement after enhanced scanning, and the CT value of the region is approximately 26 HU, and corresponds to the region indicated by the blue arrow. **(1-C1)** Thin-section transverse CT scan of the venous phase following contrast enhancement: the CT value of the hypodense region within the tumor lesion is approximately −21 HU, and corresponds to the region indicated by the blue arrow. **(1-C2)** Thin-section transverse CT scan of the venous phase following contrast enhancement: upon magnification, the CT value of the mural nodule of the tumor cyst wall is 44 HU, and corresponds to the region indicated by the blue arrow.

A 54-year-old male patient was undergoing a routine upper abdominal plain scan combined with enhanced CT at Mian Yang Wanjiang Eye Hospital. A roundish low-density space-occupying lesion was found in the left upper abdominal cavity (the space between the spleen, stomach and pancreas), approximately 3.08 cm × 2.82 cm × 2.82 cm in size. The CT values of the tumor are 12 HU, 18 HU, and 21 HU on high-resolution non-contrast CT images (Figure 1-A1),the arterial phase images after contrast enhancement (Figure 1-B1), and the portal venous phase after contrast enhancement (Figure 1-C1), respectively. The majority of its boundary was clear, while the boundary with the tail of the pancreas was partially indistinct. A wall nodule with a diameter of about 0.6 cm was visible at the anterior margin of the lesion, which showed significant enhancement after enhanced scanning (Figures 1-A2,B2,C2). No definite gas density shadow was observed in the remaining area. The imaging findings suggested a cystic tumor of pancreatic origin, but it was difficult to determine whether it was benign or malignant. For further diagnosis and treatment, the patient was admitted to the Department of Hepatobiliary and Pancreatic Surgery at Mianyang Central Hospital with a diagnosis of “intra-abdominal tumor” in the outpatient department. The patient has a history of rheumatoid arthritis for over 10 years and has been treated with leflunomide, methotrexate and folic acid for a long time. He claims that his condition is well-controlled. More than 30 years ago, he underwent an open appendectomy for acute appendicitis at a local hospital. He denies any history of other systemic diseases. He has an occasional smoking history but no other bad habits. He denies any other personal history. The results of the liver and gallbladder tumor marker tests show that the serum ferritin (FERR) is 184.72 ng/mL, carcinoembryonic antigen (CEA) is 1.39 ng/mL, alpha-fetoprotein (AFP) is 2.96 ng/mL, and CA19-9 is 9.28 U/mL. All the indicators are within the normal reference range. After a multidisciplinary consultation, it was decided to perform laparoscopic exploration and resection of the intra-abdominal mass under general anesthesia, with a plan to also carry out a distal pancreatectomy and splenectomy if necessary. Laparoscopic exploration revealed no ascites in the abdominal cavity, and no obvious abnormalities were found in the abdominal and pelvic cavities. The liver texture was normal, and no definite space-occupying lesions were observed. The spleen was morphologically normal. A cystic and solid space-occupying lesion, approximately 3.5 cm × 3.0 cm in size, was visible in the upper left abdomen, located in front of the splenic hilum and behind the stomach. The mass exhibited dense adhesion to the pancreatic tail tissue, with no distinct boundary identified; the extrapancreatic portion of the tumor was encapsulated by an intact capsule. The tumor had no adhesion with the liver, duodenum, spleen or bile duct. Based on this, the surgical plan was adjusted to laparoscopic pancreatic lesion resection combined with percutaneous abdominal drainage. The postoperative specimens were sent for pathological examination. The pathological diagnosis was: cyst, lined with stratified squamous epithelium, with sebaceous gland tissue visible in some areas, and containing many lymph nodes in the cyst wall, consistent with dermoid cyst (i.e., mature cystic teratoma). He recovered well after the operation. Nine months after the operation, he was admitted to the Mian Yang Wanjiang Eye Hospital for a follow-up examination. An abdominal CT scan showed no abnormal mass in the original lesion area in the upper left abdomen, but a linear high-density shadow was seen in the tail of the pancreas, suggesting residual metal clips after the operation.

## Discussion

Dermoid cysts are congenital developmental anomalies of germ cell origin from the ectoderm, endoderm, and mesoderm. They most commonly occur within the gonads, but have been reported in the skull, brain, mediastinum, adrenal glands, prostate, omentum, peritoneal localization (mesocolic) and retroperitoneum (11, 14, 15). The pancreas is an extremely rare primary site. As true cysts, dermoid cysts are usually benign and well-differentiated lesions. Pancreatic teratomas may originate from abnormal germ cells that are blocked from migrating to the gonads during early embryonic development. Pancreatic cystic teratomas are extremely rare, with a slightly higher incidence in females and are more common in younger individuals (Table 2). In this case, the lesion was located in the tail of the pancreas, and the patient was 54 years old, and reports of cases occurring in the tail of the pancreas in this age group are relatively rare.

**TABLE 2:** Analysis and summary of the characteristics of pancreatic dermoid cysts based on previous literature reports.

Characteristic	Number (%)
Age (mean ± standard deviation)	36.9 ± 21.8 (4 months–74 years-old)
Sex (F/M)	29:25
Symptom	54 (100%)
Abdominal pain	24 (44%)
Abdominal mass	10 (19%)
Asymptomatic	14 (25.9%)
Emesis	5 (9%)
Back pain	3 (6%)
Location	51 (100%)
Head	16 (31%)
Body	15 (29%)
Tail	12 (24%)
Body-tail	4 (8%)
Head-body	3 (6%)
No connection to the pancreas	1 (2%)
Maximum diameter (mean ± standard deviation)	9.2 ± 5.1 (2.5–22) cm
Surgery	53 (100%)
Pancreatic cystectomy	20 (38%)
SPG	9 (17%)
Distal pancreatectomy	8 (15%)
DPC	3 (6%)
PPPD	2 (4%)
Others	11 (21%)
Recurrence or metastasis or malignant transformation	25 (100%)
No	21 (84%)
Malignant transformation	0
Recurrence	2 (8%)
Neuroendocrine tumor occurred	1 (4%)
Metastasis	0
Died of surgical complications	1 (4%)

SPG, splenopancreatectomic gauche; DPC, duedunopancreatectomy cephalic; PPPD, pylorus preserving pancreaticoduodenectomy.

The diagnosis of pancreatic teratomas mainly relies on postoperative pathological examination, as its clinical manifestations and laboratory tests lack specificity. Imaging examinations often misdiagnose it as primary cystic tumors of the pancreas, and this case is no exception. In the differential diagnosis of mature teratoma, immature teratoma and primary benign and malignant pancreatic tumors, laboratory examination indicators such as CA 19-9, CA 125, CA 72-4, CEA, AFP, and HCG have certain clinical value (16). However, the tumor markers mentioned above were within normal range, thus making it difficult to achieve effective differentiation through laboratory tests. The features observed in imaging examination images are influenced by the relative quantities of different tissue types within the lesion, including fat, lipid-fluid levels, and calcification (17). When a pancreatic dermoid cyst is suspected, several differential diagnosis should be considered such as pancreatic pseudocysts, serous cystadenomas, mucinous cystadenomas, solid pseudopapillary tumors (SPT), epidermoid cysts of accessory spleen within the pancreas (ECIPAS), and lymphoepithelial cysts (LECs). The accurate differentiation of these diseases is crucial for correct diagnosis and treatment. In this case, the lesion exhibits predominantly well-defined margins, with only the posterior border showing partial indistinctness from the pancreatic tail parenchyma. Mucinous cystic neoplasms of the pancreas are strictly confined within the pancreatic parenchyma, while mature teratomas in the abdominal cavity can be adjacent to the pancreas. The presence of calcification further supports the possibility of a mature teratoma as the diagnosis. However, in our case, no calcification or air-fluid levels were identified within the pancreatic mature cystic teratoma. Its CT value lies between the gastric contents and pure fat. The density within the cyst is uniform. The imaging manifestations lack typical features of teratoma, suggesting that it is difficult to clearly distinguish it from pancreatic-derived cystic tumors in preoperative imaging differential diagnosis.

All abdominal dermoid cysts warrant surgical resection due to the risk of spontaneous rupture, and chemical peritonitis caused by rupture is difficult to treat and may require repeated laparotomies (11). Surgical resection is the main treatment for pancreatic teratoma, but there is currently a lack of comprehensive surgical guidelines. Ideally, complete resection without damaging important structures is crucial, as incomplete resection usually leads to poor oncological outcomes. Choosing the appropriate surgical approach based on the tumor location and achieving complete resection is the gold standard for diagnosis and treatment (18). In this case, the tumor exhibited a predominantly intact capsule, with only focal areas showing dense adhesion to the pancreatic tail parenchyma. Successful en bloc resection was achieved intraoperatively without complications. Therefore, there was no need to perform a distal pancreatectomy. This not only better preserved the pancreatic function but also reduced the surgical trauma and was conducive to postoperative recovery.

Malignant transformation of mature cystic teratoma is a rare complication with an incidence of 1%–2%, especially in postmenopausal women (19). Magnetic Resonance Imaging (MRI) demonstrates superior utility in evaluating the benign-malignant potential of masses. The presence of imaging features including invasive growth, irregular margins, capsular breach, marked post-contrast enhancement, and restricted diffusion warrants a high index of suspicion for malignant lesions (20). In post-pubertal males, mature teratomas are considered malignant due to their derivation from transformed germ cell elements and their potential for recurrence, local invasion, and malignant transformation (21). Accordingly, for patients with residual teratoma lesions, active surgical intervention and long-term surveillance remain clinically significant, even in the setting of normal-range tumor marker levels. In this case, the patient achieved total resection of the tumor, with a relatively low risk of local recurrence and malignant transformation. Furthermore, in previously published cases of pancreatic dermoid cysts, the recurrence rate was 8% (2/25), the rate of neuroendocrine tumor transformation was 4% (1/25), and no cases of malignant transformation were documented, indicating a favorable overall prognosis for this tumor entity (Table 2). The patient was followed up for 10 months after the operation, and the general condition was good, with no signs of malignant transformation. Long-term follow-up is still needed in the future.

This case highlights the challenges in the imaging diagnosis of mature cystic teratoma of the pancreas, and emphasizes the significant value of the imaging examination results and the intraoperative clinical observation and cognition of the tumor by the surgeon in guiding the formulation of the surgical plan. CT imaging diagnosed it as a primary cystic tumor of the pancreas, while postoperative pathological examination confirmed it as a mature cystic teratoma. It indicates the necessity of comprehensive preoperative and intraoperative assessment to avoid unnecessary expansion of the surgical scope.

## Conclusion

This study reports a rare case of a mature cystic teratoma in the tail of the pancreas. Although the patient had no obvious clinical symptoms, imaging examinations suggested a primary cystic tumor in the tail of the pancreas. Based on the intraoperative clinical observation and judgment of the tumor by the surgeon, a laparoscopic resection of the pancreatic mass was performed. This case highlights the crucial role of thorough preoperative imaging assessment and intraoperative clinical decision-making in managing such rare and diagnostically challenging diseases. It also reflects the diagnostic difficulties posed by the non-specific imaging features of the tumor and its high similarity to common pancreatic cystic lesions. In conclusion, for pancreatic cystic lesions that lack radiological evidence of calcification and fat components, the possibility of mature cystic teratoma should still be taken into consideration in the differential diagnosis.

## Data Availability

The original contributions presented in this study are included in this article/supplementary material, further inquiries can be directed to the corresponding authors.
